# Toward Portable and Affordable Air Quality Monitoring: A 3D-Printed Platform for Colorimetric NO_2_ Quantification from Vehicles’ Exhaust Emissions

**DOI:** 10.1021/acsomega.5c10914

**Published:** 2026-02-05

**Authors:** Danielle da Silva Sousa, Sidnei Gonçalves da Silva, João Flávio da Silveira Petruci

**Affiliations:** Institute of Chemistry, 28119 Federal University of Uberlândia (UFU), 38408-072 Uberlândia, Minas Gerais, Brazil

## Abstract

Air pollution from vehicular exhaust emissions remains one of the main environmental and public health concerns in urban areas. Nitrogen dioxide (NO_2_), a major component of combustion-related pollutants, is of particular concern due to its harmful effects and contribution to secondary atmospheric reactions. Its emissions from engines are continuously monitored to mitigate pollution and to support technological advances in vehicle energy sources. For this purpose, suitable sensor technologies must be developed to enable in situ analysis with ease of operation and compact design. This study presents the development and validation of a portable analytical platform designed for the quantification of gaseous NO_2_ directly from vehicle exhaust emissions. The system was assembled using commercially available components and 3D-printed parts, employing a colorimetric detection method based on the Griess–Saltzman reaction combined with an ESP32-S3 microcontroller responsible for data acquisition and signal processing. Analytical parameters, such as sampling time and flow rate, were optimized to improve the efficiency of the NO_2_ capture. The developed platform achieved a limit of detection of 0.6 ppbv and a limit of quantification of 2 ppbv for a sampling time of 20 min, ensuring high sensitivity for trace-level detection. Application of the device in real vehicle exhaust analysis revealed significant variations among fuel types, with diesel vehicles exhibiting NO_2_ emissions substantially higher than those of gasoline-fueled ones. The results demonstrate the feasibility of using low-cost, portable systems for NO_2_ monitoring, supporting their potential use in preliminary air quality assessments and urban pollution source identification.

## Graphical abstract

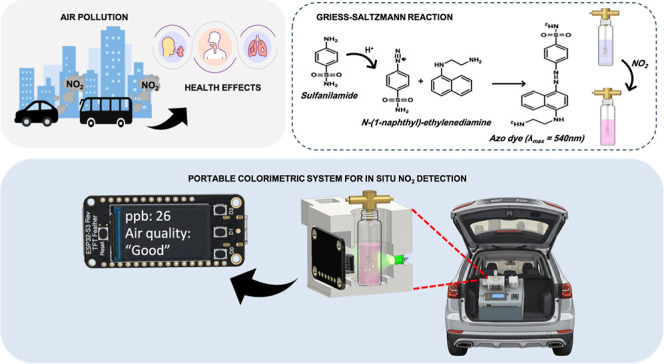

## Introduction

Air pollution in urban environments has emerged as one of the most pressing environmental and public health concerns worldwide.^1^ Rapid urbanization, population growth, and the increasing demand for mobility have intensified emissions from on-road vehicles, making traffic a dominant source of atmospheric pollutants in metropolitan areas.^2^ Among these pollutants, nitrogen oxides (NO_*x*_ = NO and NO_2_) stand out due to their prevalence, atmospheric reactivity, and well-documented health impacts.^3^

Nitrogen oxides emitted by on-road vehicles are major contributors to urban air pollution. Although diesel vehicles currently account for only a small fraction of the total traffic fleet, they are responsible for a disproportionate share of NO_*x*_ emissions with freight trucks being the dominant source. The fraction of NO_*x*_ emitted directly as nitrogen dioxide (NO_2_) is particularly concerning, as it has been consistently associated with elevated risks of respiratory and cardiovascular health effects in epidemiological studies.^4^ In areas with elevated emissions of volatile organic compounds (VOCs), NO_2_ can persist longer in the atmosphere, allowing it to be transported over extended distances and contributing to photochemical pollution events far from the emission sources.^5–7^ Therefore, the presence of NO_*x*_ in the atmosphere, in conjunction with VOCs and solar radiation, leads to the formation of ozone, a major secondary pollutant in the troposphere.

Urban areas are hotspots for air pollution due to dense population and the high concentration of on-road vehicles. Air quality in these metropolitan environments is highly heterogeneous, exhibiting complex spatial and temporal patterns across citywide, urban–rural, and neighborhood scales. Pollutant levels can also display highly localized and transient peaks near major roadways and traffic hubs, highlighting the dominant influence of vehicular emissions and the need for monitoring approaches capable of capturing fine-scale variations in pollutant levels.^8^

Therefore, the emissions of NO_2_ by vehicles are controlled by specific national and international legislations. There are emission standards related to NO_*x*_ for different types of vehicles (i.e., cars and light trucks) and fuels (i.e., gasoline and diesel) established by Brazil, the European Union, and the United States. In the US, the Tier 3 program establishes a fleet-average limit of 0.03 g/mile (∼0.019 g/km) of NO_*x*_ + NMOG (non-methane organic compounds) for light-duty vehicles.^9^ In the European Union, the upcoming Euro 7 standards set limits of 0.06 g/km for gasoline vehicles and 0.08 g/km for diesel vehicles.^10^ In Brazil, PROCONVE L-8 imposes a limit of 0.050 g/km for passenger cars and 0.140 g/km for light commercial vehicles, with progressive reductions planned in the coming years.^11^ However, even with strict regulatory emission tests, some vehicles were found to activate emission control systems only during testing, meeting legal limits while emitting significantly higher NO_*x*_ under real-world driving conditions. Nevertheless, many diesel vehicles exceed legal limits; in Brazil, 57% of heavy-duty diesel vehicles failed to comply with the PROCONVE standard.^12^ Globally, analyses of 11 major markets—covering over 80% of diesel vehicle—estimated 13.1 million tons of NO_*x*_ emissions under real conditions, exceeding laboratory predictions by 4.6 million tons.^13^

In such scenarios, the quantification of the NO_2_ emissions from vehicles should rely on analytical methods capable of providing reliable and in situ results. Generally, gaseous NO_2_ quantification has been performed using various approaches, resulting in both direct and indirect analytical methods with differing limits of detection, selectivity, and operational feasibility, depending on the intended application.^8,14^ The chemiluminescence-based method remains widely used as the reference technique for the NO measurement. For NO_2_ quantification, the most common approach involves its conversion to NO through heated molybdenum surfaces or UV photolysis systems, allowing indirect measurement via the same principle.^15^ However, these methods may be affected by reactive compounds present in ambient air, such as nitrous acid (HONO), nitric acid (HNO_3_), and peroxyacetyl nitrate (PAN), which can also be converted to NO, compromising selectivity and accuracy.^16^ Furthermore, the large footprint of the instrumentation and the high associated costs hinder its suitability for routine deployment.

Gas sensors based on metal oxide semiconductors (MOX) rely on surface redox reactions and are recognized for combining several practical advantages, including low production cost, elevated sensitivity, quick response, and straightforward integration into electronic systems. These features make them promising candidates for portable and field-deployable applications.^17^ However, they still present critical limitations, including elevated power demand, restricted sensitivity in trace-level detection, delayed response/recovery behavior, vulnerability to environmental conditions (e.g., humidity), and lack of long-term stability.^18^ Colorimetric methods—usually using the well-recognized Griess–Saltzman reaction—have been extensively employed for low-cost NO_2_ quantification by combining several approaches including passive sampling followed by digital image treatment or portable platforms that combine sample collection with subsequent colorimetric analysis.^19–22^ Also, devices with high spatiotemporal resolution have been proposed.^23–26^ Although previous studies have demonstrated inherent selectivity and satisfactory sensitivity, they often lack either online analytical capability (e.g., when passive sampling is employed) or integrated data acquisition and transmission functions, which limits their applicability in real-world scenarios.

In this study, we have designed and validated a NO_2_ monitoring system with low-cost, commercially available components using an ESP32 microcontroller for both data acquisition and processing. The analytical method is based on the Griess–Saltzman reaction, where NO_2_ from the air reacts with the reagent to produce a distinct color change that can be quantified.^27,28^ Custom parts necessary for assembly were produced with a 3D printer, while the control and data processing are handled by firmware developed internally, ensuring low cost and adaptability for future improvements. Despite relying on a reagent-based capture approach, the integration with the ESP32 platform provides a practical compromise between affordability, simplicity of construction, ease of use, and spatiotemporal monitoring capability. The system allows periodic measurements, providing flexibility for deployment across multiple locations. The low production cost and computational capabilities of ESP32 further enable the potential expansion of multiple devices into a network for broader, city-scale air quality monitoring.

## Materials and Methods

### Reagent Preparation

The Griess–Saltzman reagent was prepared by dissolving 2.5 g of sulfanilic acid (Carlo Erba, Cornaredo, Italy) in 300 mL of deionized water (GEHAKA purification system, São Paulo, Brazil), followed by the addition of 70 mL of glacial acetic acid (Maia, São Paulo, Brazil) and 10 mg of *N*-(1-naphthyl)­ethylenediamine dihydrochloride (Merck, Kenilworth, NJ, USA). The final volume was adjusted to 500 mL with deionized water.

For optimization and calibration of the analytical platform, standard gaseous NO_2_ mixtures were generated by using a permeation method system. Compressed air was purified by passing it through two sequential columns filled with silica gel and potassium iodide (KI). The purified air was then directed through a custom-built glass chamber containing a NO_2_ permeation tube (VICI Metronics, Santa Clara, CA, USA) with a certified permeation rate of 81.25 ng min^–1^ at a constant temperature of 35 °C. Adjusting the airflow from 0.3 to 2.0 L min^–1^ generated NO_2_ concentrations ranging from 27 to 180 ppbv.

### Analytical Platform for Gas Monitoring

The device housing was designed by three-dimensional modeling and fabricated by 3D printing with PLA filament (GTMax3D Core A2v2). The design ensured proper alignment of optical components and ambient light shielding to minimize interferences. The internal compartment housed the battery and flow regulation system, while the main module contained the ESP32-S3 with a display, the sensor, and the vacuum mini pump.

The portable photometric system was operated using an ESP32-S3 microcontroller (Adafruit Feather ESP32-S3 Reverse TFT, USA) with an integrated 1.14 in. color TFT display (240 × 135 px). The module also included a TSL2591 digital light sensor (Adafruit, USA) configured with a 25× gain and an integration time of 200 ms and a green LED (λ = 540 nm), positioned on opposite sides of a glass vial with an optical path of ∼1 cm for transmittance detection. The LED used was an Adafruit LED Sequin, which incorporates a 100 Ω current-limiting resistor, drawing approximately 5 mA at 3.3 V. Power supply was provided by a 3.7 V, 5000 mAh rechargeable lithium-ion battery or USB-C connection. Gas sampling was performed by using a miniature air pump (45 × 21 × 12 mm, 3 V) connected to PTFE tubing and the vial containing the reagent solution. Pump activation was controlled by an on/off switch wired to the ESP32-S3 board. Figure 1 presents the 3D rendered model of the proposed analytical platform for NO_2_ quantification.

**Figure 1:**
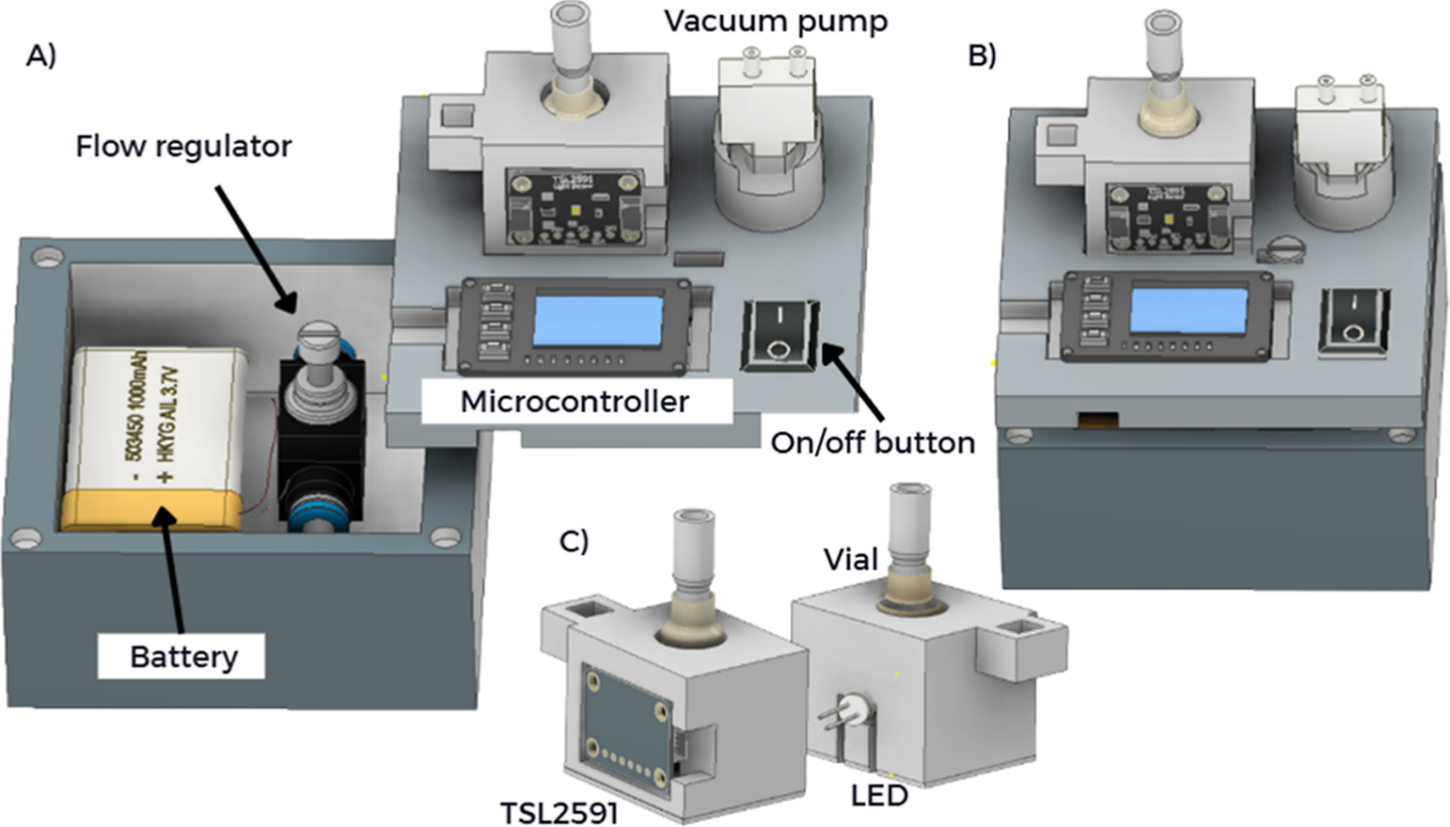
Portable photometric platform developed for NO_2_ detection in vehicle exhausting systems. Main components: (A) battery, flow regulator, microcontroller, and vacuum pump; (B) overall view of the assembled platform; and (C) TSL2591 sensor coupled to the vial with LED.

### Analysis Protocol

The analysis process begins by pressing the D0 button on the ESP32 microcontroller and manually activating the vacuum mini pump via the switch button, which promotes the bubbling of atmospheric air into a vial containing 1000 μL of the Griess–Saltzman reagent. A 2 mm diameter Teflon tube is inserted into the vial, allowing small air bubbles (approximately 2 mm) to be directly introduced into the reagent, thereby enhancing the reaction efficiency. During sampling, the mini pump maintains a constant flow of 50 mL min^–1^ for a programmed period of 20 min, with the flow being regulated by a control valve (Figure 2). At the end of this interval, the TSL2591 digital sensor measures the final light intensity (*I*), expressed in lux. Figure 3 presents a schematic representation of the introduction of vehicle exhaust air into a vial containing the reagent.

**Figure 2:**
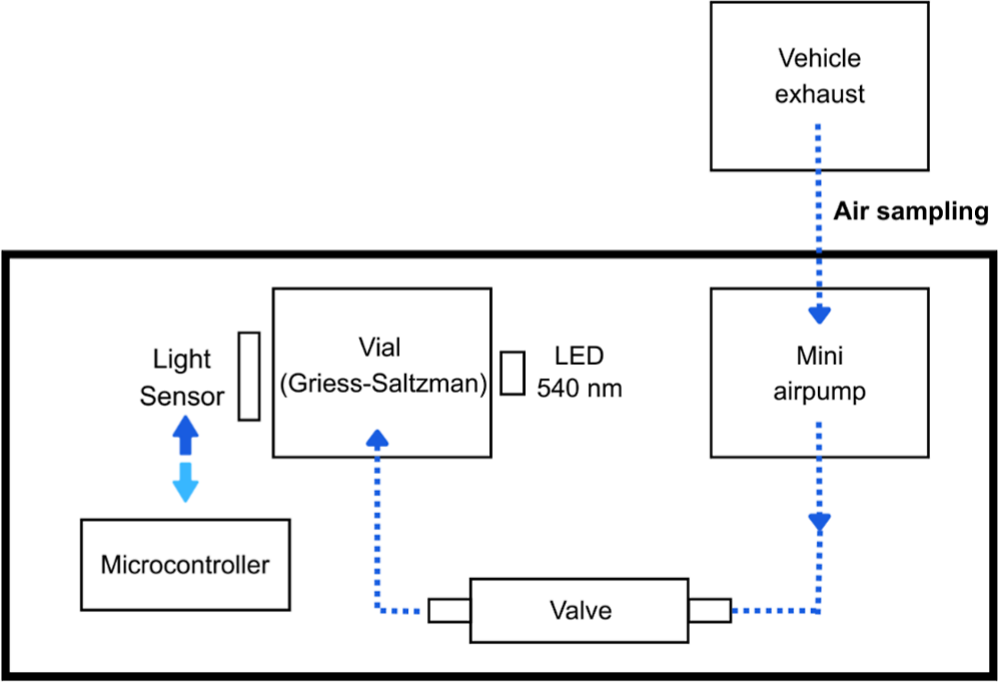
developed analytical platform for NO_2_ quantification is schematically represented.

**Figure 3:**
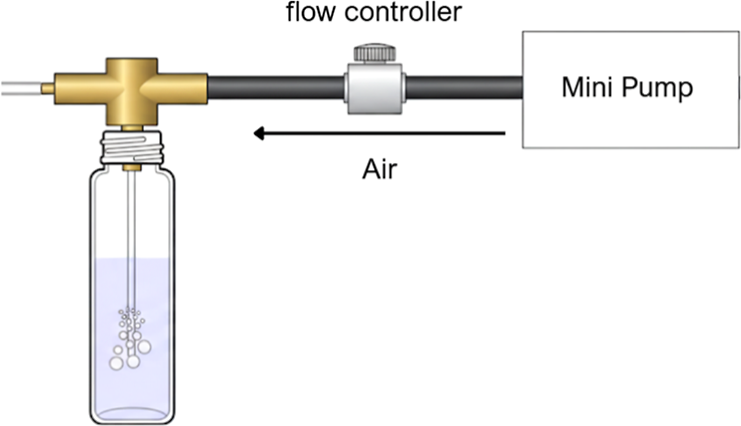
Schematic of vehicle exhaust air introduction into a vial containing the Griess–Saltzman reagent via a mini pump, with a controlled flow of 50 mL min^–1^, showing air bubbling through the liquid for the reaction of the NO_2_ present.

The light intensity signal is processed directly by the firmware embedded in the ESP32, which calculates absorbance based on the initial intensity obtained from the blank and the final intensity of the sample, according to eq 1. Since sequential acquisition of the blank and sample signals was not feasible without interrupting the measurement when replacing the reagent, the blank intensity (*I*_0_) was determined prior to the analyses. For this purpose, multiple blank measurements were performed under identical operating conditions and the resulting values were averaged to obtain a stable reference signal. This average blank intensity (*I*_0_ = 541) was then implemented as a fixed value in the firmware for all calculations. The differences in light intensity (Δ*I*) were used as the primary analytical output. Absorbance was calculated by comparing the average sample signal to the blank using the equation

1 $$Absorbance=\log\frac{I_{0}}{I}$$

where *I* represents the average light intensity of the sample in lux and *I*_0_ corresponds to the blank signal in lux. All measurements were performed in triplicate to ensure the reproducibility of the results.

Subsequently, the absorbance is converted to the concentration using the analytical calibration curve previously incorporated into the code. The result, expressed in parts per billion by volume, is automatically displayed on the device screen. The complete code configuration used to obtain these results is provided in the Supporting Information.

## Results and Discussion

### Evaluation of the Sampling Time and Flow Rate

The analytical method relies on air sampling by bubbling the air stream directly through the reagent solution, enabling a subsequent chemical reaction between the analyte (NO_2_^–^) and the Griess–Saltzman reagent. The efficiency of the transference of the analyte to the reagent solution determines key parameters, such as the limit of detection, response time, and linear dynamic range. Initially, the influence of the sampling time on the analytical signal was assessed for 20 and 30 min, while maintaining a constant bubbling flow of 100 mL min^–1^. Gaseous NO_2_ standard with a concentration of 36 ppbv and a reagent volume of 800 μL were employed. The analytical signal—as described in the Materials and Methods—was obtained for each sampling time. The comparison of these sampling periods indicated that increasing the sampling time for 10 min significantly enhanced the amount of NO_2_ captured (Figure 4a).

**Figure 4:**
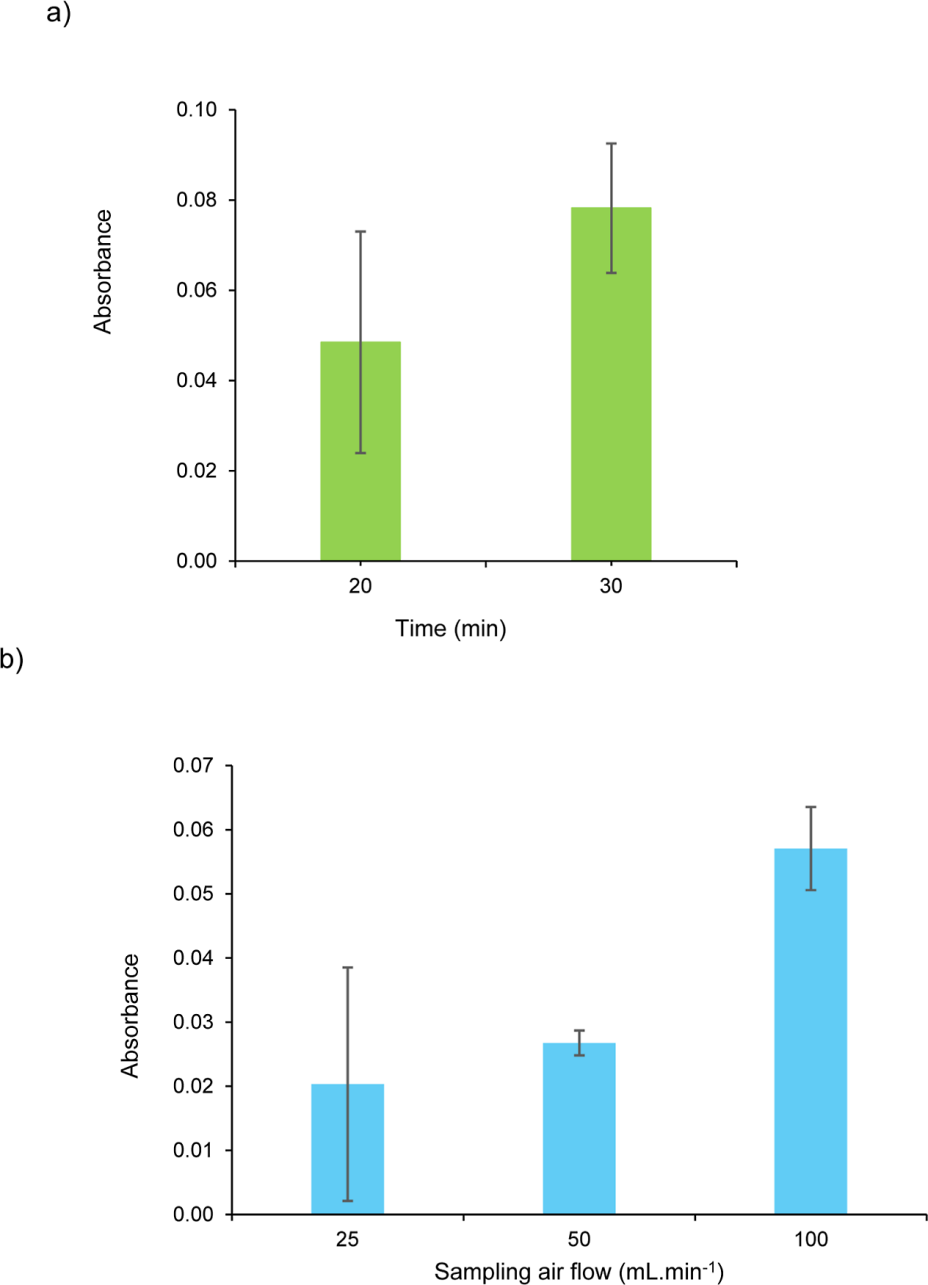
(a) Variation of the analytical signal as a function of sampling time (20 and 30 min). (b) Effect of sampling airflow (25, 50, and 100 mL min^–1^) on the analytical signal, obtained using a fixed sampling time of 20 min.

Next, the effect of the sampling flow rate on the analytical signal was evaluated at 25, 50, and 100 mL min^–1^ under a fixed sampling time of 20 min, with a NO_2_ concentration of 36 ppbv and reagent volume of 800 μL. As can be seen in Figure 4b, the highest average absorbance was obtained at 100 mL min^–1^, indicating maximum NO_2_ capture. However, this flow might cause reagent loss due to excessive bubbling, compromising the analytical reliability. Conversely, the lowest flow (25 mL min^–1^) exhibited a high variability (89.6%), likely due to operating near the lower limit of the rotameter, which increased susceptibility to flow fluctuations. Consequently, a flow of 50 mL min^–1^ was selected as optimal, balancing efficient NO_2_ capture with system stability.

### Evaluation of Reagent Solution Saturation

Despite the simplicity of the colorimetric approach, an inherent drawback is the need to replace the reagent solution before each analysis. In this study, we performed an additional experiment to evaluate potential reagent saturation over time with the aim of reusing the same solution for multiple analyses. Continuous sampling was performed over 3 h, with absorbance measured every 20 min. Experimental conditions were fixed with a flow rate of 50 mL min^–1^, NO_2_ concentration of 36 ppbv, and a reagent volume of 800 μL. The air sampling was maintained continuously without reagent replacement, allowing assessment of both analytical response variation and reagent capacity under prolonged exposure. The increase in absorbance over time is shown in Figure 5. A linear behavior of the analytical signal was observed over seven consecutive sampling procedures, corresponding to 140 min of air bubbling. This result demonstrates the capability of the sampling method to use the same reagent solution repeatedly, thereby facilitating its application in real-world monitoring scenarios.

**Figure 5:**
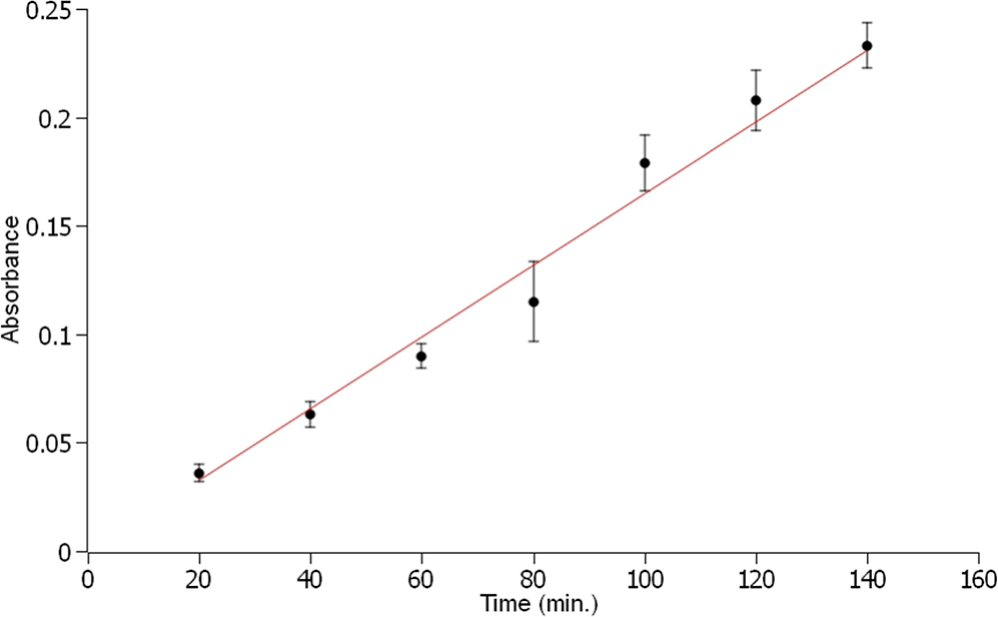
Variation of absorbance during continuous sampling over approximately 3 h at a sampling rate of 50 mL min^–1^.

### Analytical Figures-of-Merit

For quantitative purposes, an analytical calibration curve was obtained by plotting the average of three absorbance measurements against gaseous NO_2_ standard solutions prepared by the permeation method. A sampling airflow of 50 mL min^–1^ for 20 min was used, with the gas bubbled into a flask containing 800 μL of the reagent solution. Linear relationships between the analytical signal and concentration were built in the range of 27 to 180 ppbv. It is important to note that the lower limit of the linear range could, in principle, be decreased; however, due to limitations of the permeation tube and the airflow meters, our system was not able to generate lower concentrations. Nevertheless, for the intended application, this concentration range is suitable.

The limits of detection (LOD) and quantification (LOQ) were calculated as three and ten times the standard deviation of the blank (*n* = 9), yielding 0.6 and 2.0 ppbv, respectively. The validation results and optimized conditions are summarized in Table 1.

**Table 1:** Analytical Parameters for NO_2_ Quantification and Optimized System Conditions

parameter	values
linear range	27–180 ppbv
equation	*A* = 0.0064[NO_2_] – 0.11
linearity (*R*)	0.994
LOD (ppbv)	0.6
LOQ (ppbv)	2.0
sampling airflow	50 mL min^–1^
sampling time	20 min

### Evaluation of Accuracy via Recovery Tests

Some potential interferences associated with the Griess–Saltzman reaction have been described in the literature. Ambient HONO is known to react with sulfanilamide and may contribute to a positive bias; however, its interference is typically very small. With respect to other atmospheric species, ISO 6768 reports that nitrogen monoxide, hydrogen sulfide, hydrogen chloride, fluorine compounds, and typical levels of SO_2_ do not interfere with the determination of the concentration of NO_2_. Additional studies^20^ found that most common gases present in polluted air cause negligible interference, with the exception of SO_2_ at very high SO_2_/NO_2_ ratios (30:1), which may slowly bleach the azo dye. Considering these findings, the main known interferents either occur at negligible concentrations or introduce significant bias only under conditions that differ substantially from those used in this study.

To assess the accuracy of the developed platform, a recovery test was carried out using a standard gas solution employing a certified NO_2_ permeation tube, as described in the Materials and Methods. Air analysis was performed using the optimized experimental conditions and the obtained concentrations were compared with the reference (35 ppbv). The mean concentration of 32 ± 0.7 ppbv was achieved, resulting in a recovery value of 91%. According to the U.S. EPA Enhanced Air Sensor Guidebook,^29,30^ low-cost sensors (<USD2500) are considered suitable for exploratory and citizen science applications even with errors up to 50% (Tier I—education/awareness), and for hotspot identification with errors ≤30% (Tier II). Therefore, the results indicate that the platform’s performance is consistent with preliminary environmental mapping and educational monitoring applications, demonstrating the system’s ability to reliably detect NO_2_ at concentrations typical of monitored environments.

### Application of the Proposed Platform for NO_2_ Monitoring in Vehicle Exhaust Emissions

The developed analytical platform was applied in a preliminary field study to quantify NO_2_ directly from motor vehicle exhaust emissions. Exhaust gases were sampled from six light-duty vehicles operating on gasoline/ethanol blends and one diesel-powered vehicle. For these experiments, the platform was positioned in the trunk of each vehicle and connected to a Teflon tube inserted directly into the exhaust outlet (Figure 6a). The tube was coupled to the mini vacuum pump, which maintained a controlled gas flow of 50 mL min^–1^ for a sampling period of 20 min. The concentration data were displayed on the device screen during the experiments (Figure 6b). The results are presented in Table 2, where NO_2_ concentrations are reported alongside vehicle year and fuel type.

**Figure 6:**
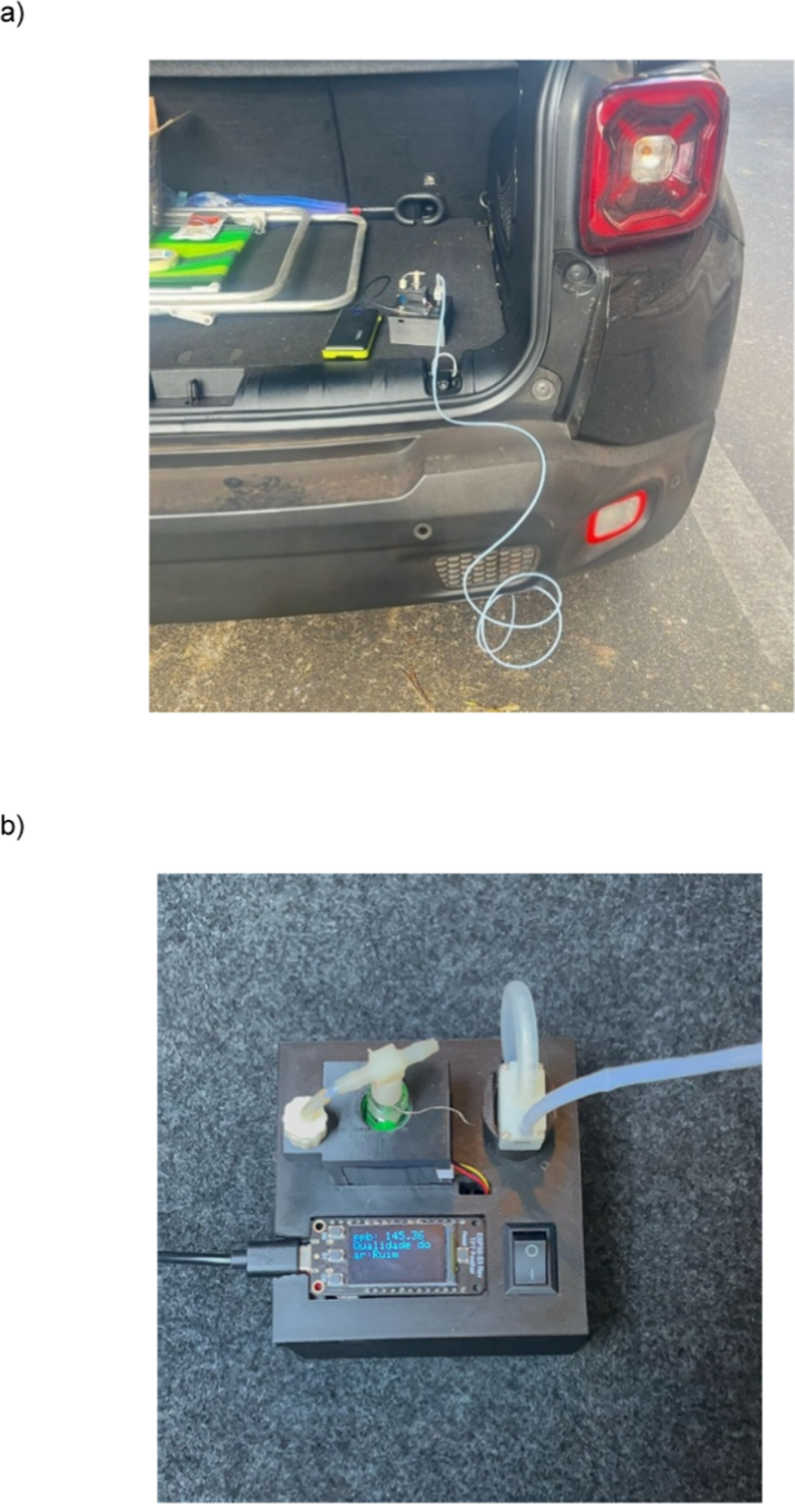
(a) Deployment of the proposed analytical platform for the monitoring of vehicle exhaust emissions and (b) top view of the platform.

**Table 2:** NO_2_ Concentrations Measured in Six Vehicles of Different Models and Fuel Types Using the Developed Analytical Platform

vehicle	fabrication year	fuel	concentration (ppbv)
vehicle 1	2015	gasoline	43 ± 13
vehicle 2	2025	gasoline	112 ± 42
vehicle 3	2020	diesel	940 ± 65
vehicle 4	2018	gasoline	185 ± 5
vehicle 5	2018	gasoline	116 ± 15
vehicle 6	2024	gasoline	78 ± 1

As expected, the diesel-powered vehicle displayed markedly higher NO_2_ emissions compared with gasoline-fueled vehicles. Variations among gasoline vehicles are attributed to differences in the ethanol content of the blended fuel, the efficiency of catalytic converter systems, and potentially the origin or quality of the gasoline used.

Multiple operational factors directly influence the formation and speciation of nitrogen oxides (NO_*x*_) in vehicle exhaust, significantly modifying the fraction of nitrogen dioxide (NO_2_) immediately prior to sampling. Under low ambient temperature conditions, thermal decreases in the engine and catalytic systems substantially increase NO_*x*_ emissions and alter the NO_2_/NO_*x*_ ratio, indicating that the oxidation of NO to NO_2_ becomes less efficient both during the initial stages of combustion and within post-treatment reactions.^31^ Complementarily, during the cold-start phase, both gasoline and diesel engines exhibit transient increases in NO_*x*_ due to delayed heating of catalytic converters and NO_*x*_ reduction systems, which postpone the reactions responsible for the efficient conversion of NO into NO_2_.

Beyond external thermal influences, internal engine conditions also modulate the NO/NO_2_ speciation. The NO_2_/NO_*x*_ fraction decreases under high-load conditions, as elevated in-cylinder temperatures shift the chemical equilibrium toward NO formation.^32^ Also, oxygen availability plays a determining role: mixtures with lower O_2_ content suppress NO_2_ formation even when NO is already present, whereas more oxidizing conditions partially favor its conversion.

Even under seemingly stable conditions—such as continuous idling without acceleration—small natural fluctuations in exhaust temperature can affect the rate of NO oxidation to NO_2_, modifying the observed speciation over time.^33^ Thus, within the context of the present study, it is plausible that the gradual thermal evolution during the 20 min engine operation resulted in minor variations in the NO_2_ concentration, even in the absence of external changes in fuel, ambient conditions, or engine operation. This behavior is inherent to the chemical dynamics of vehicular exhaust and does not compromise the applicability of the method but reinforces the need to interpret the results considering that the NO/NO_2_ equilibrium is thermally and operationally sensitive even under controlled conditions.

## Conclusion

Here, we propose the fabrication of a portable analytical platform for the quantification of gaseous NO_2_ directly from vehicle exhaust emissions. The platform was fabricated using FDM 3D printing and was based on low-cost components, such as LED, mini pump, power bank, and a microcontroller. The instrument demonstrated promising results in terms of sensitivity, stability, and practical applicability. Optimization of the experimental parameters, particularly the controlled flow rate of 50 mL min^–1^ and a sampling time of 20 min, significantly improved the efficiency of analyte capture.

Application of the system to real exhaust gas samples confirmed its ability to detect variations in NO_2_ concentrations across different vehicles, reflecting its potential for exploratory monitoring in urban environments. As expected, the diesel-powered vehicle exhibited substantially higher NO_2_ levels compared with gasoline vehicles, in agreement with the known emission profiles of these fuel types. While wide-range accuracy constraints must be acknowledged, the method developed in this work offers a practical and accessible alternative for field assessment of NO_2_, with potential applications in the identification of localized atmospheric pollution sources and for deployment in diverse monitoring scenarios, including parking lots and both indoor and outdoor environments.

## Supplementary Material



## Notes

Customized Arduino code developed for this study (PDF)
