# Supplementary material for: Toward Portable and Affordable Air Quality Monitoring: A 3D-Printed Platform for Colorimetric NO2 Quantification from Vehicles’ Exhaust Emissions
Source: ACS Omega. 2026 Feb 5;11(6):10030–7. doi: 10.1021/acsomega.5c10914 (PMC12917802; doi:10.1021/acsomega.5c10914)
Supplement: Supplementary file 1 [file ao5c10914_si_001.pdf]

SUPPLEMENTARY INFORMATION

Towards portable and affordable air quality monitoring: a 3D-printed platform for colorimetric NO_2 quantification from vehicles exhaust emissions

Danielle da Silva Sousa, Sidnei Gonçalves da Silva and João Flávio da Silveira Petruci*

Federal University of Uberlândia (UFU), Institute of Chemistry, 38408-072, Uberlândia-MG, Brazil

***Corresponding author:** jfpetruci@gmail.com

1. Automation and Transmission of the Analytical Signal

The system was automated using a custom code developed in the Arduino IDE and executed on an ESP32 microcontroller. The code controls the TSL2591 light sensor, processes the acquired data to calculate analyte absorbance, and converts this value into an estimated concentration. Results are displayed on the ESP32 TFT screen, enabling real-time visualization.

Initially, the code includes the necessary libraries for controlling the TSL2591 sensor, the TFT display, and I²C communication. Display connection pins are defined, and variables are declared to store light intensity readings as well as absorbance and concentration calculations. The TSL2591 sensor is then instantiated for subsequent measurements.

The setup() function is executed once upon device initialization. During this stage, serial communication and the TFT display are initialized for result presentation. The TSL2591 sensor is configured with appropriate gain and integration time settings to ensure stable and accurate readings under medium light conditions.

During operation, the sensor records light intensity at defined intervals (20 minutes). Absorbance is calculated as the logarithm of the ratio between a fixed reference value (corresponding to the blank) and the measured lux. This absorbance is then converted into ppbv based on the calibration curve. The resulting concentration is displayed on the TFT screen and transmitted via the serial port when the microcontroller is connected to a computer, allowing real-time monitoring.

Concentration values are further classified according to air quality ranges defined by the Air Quality Index (AQI) under Brazilian environmental regulations (CONAMA nº 491/2018). The classifications include “Good,” “Moderate,” “Poor,” “Very Poor,” and “Extremely Poor,” which are displayed alongside the measured concentration.

Listing 1: Program developed in the Arduino IDE for ESP32-S3-TFT- feather based automated NO_2 measurement using a TSL2591 light sensor. The script records initial and final lux values, calculates absorbance, converts it to estimated ppbv concentration, displays the result on the integrated TFT screen, and classifies air quality according to CONAMA 491/2018 standards.

```
#include <Wire.h>
#include <Adafruit_Sensor.h>
#include "Adafruit_TSL2591.h"
#include <Adafruit_ESP32.h>

// Display pin definitions
#define TFT_CS   10
#define TFT_DC    9
#define TFT_RST   8
#define TFT_I2C_POWER 7

// Initialization of display and sensor
Adafruit_ST7789 tft = Adafruit_ST7789(TFT_CS, TFT_DC, TFT_RST);
Adafruit_TSL2591 tsl = Adafruit_TSL2591(2591);

// Main variables
float li;   // Initial lux (blank)
float lf;   // Final lux (after reaction)
float ABS;  // Absorbance
float ppb;  // Estimated concentration in ppbv

// Initial setup
void setup(void) {
  Serial.begin(115200);

  // Power supply for the display
  pinMode(TFT_I2C_POWER, OUTPUT);
```

```
  digitalWrite(TFT_I2C_POWER, HIGH);
  delay(10);

  // Initialize display
  tft.init(135, 240);
  tft.setRotation(3);
  tft.fillScreen(ST77XX_BLACK);
  tft.setTextSize(3);
  tft.setTextColor(ST77XX_BLUE);

  // Initialize TSL2591 sensor
  if (tsl.begin()) {
    Serial.println(F("TSL2591 sensor found!"));
  } else {
    Serial.println(F("TSL2591 sensor not detected!"));
    while (1);
  }
  // Gain and integration time configuration
  tsl.setGain(TSL2591_GAIN_MED);
  tsl.setTiming(TSL2591_INTEGRATIONTIME_200MS);
}
// Operation function: reading, calculation, and display
void OperacaoTSL() {
  // Initial reading (blank lux)
  li = tsl.calculateLux(tsl.getFullLuminosity(), tsl.getFullLuminosity() >> 16);
  Serial.print("Initial Lux: "); Serial.println(li);

  delay(1200000); // Sampling time ~20 min

  // Final reading (lux after reaction)
  lf = tsl.calculateLux(tsl.getFullLuminosity(), tsl.getFullLuminosity() >> 16);
  Serial.print("Final Lux: "); Serial.println(lf);
```

```
// Absorbance calculation and conversion to ppbv
if (If > 0) {
  ABS = log10(541 / If);
} else {
  ABS = 0;
}
ppb = (((ABS - 0.0341) / 30000000) * 24.5) * 1e9;
// Display concentration value
tft.fillScreen(ST77XX_BLACK);
tft.setCursor(0, 8);
tft.print("ppb: ");
tft.println(ppb);
Serial.print("Concentration (ppbv): ");
Serial.println(ppb);

// Air quality classification (CONAMA 491/2018)
if (ppb <= 21) {
  tft.println("Air Quality: Good");
  Serial.println("Air Quality: Good");
} else if (ppb <= 53) {
  tft.println("Air Quality: Moderate");
  Serial.println("Air Quality: Moderate");
} else if (ppb <= 80) {
  tft.println("Air Quality: Poor");
  Serial.println("Air Quality: Poor");
} else if (ppb <= 133) {
  tft.println("Air Quality: Very Poor");
  Serial.println("Air Quality: Very Poor");
} else {
  tft.println("Air Quality: Terrible");
  Serial.println("Air Quality: Terrible");
```

```
  }
  delay(2000); // Wait before next measurement
}
```
